# DAILY ARGUMENTS WITH SPOUSES AND NEGATIVE AFFECT: AGE AND EMOTION WORK AS MODERATORS

**DOI:** 10.1093/geroni/igac059.2334

**Published:** 2022-12-20

**Authors:** Dakota Witzel, Madeline Nichols, Justin White, Robert Stawski

**Affiliations:** Penn State University, State College, Pennsylvania, United States; Oregon State University, Corvallis, Oregon, United States; Oregon State University, Corvallis, Oregon, United States; Oregon State University, Corvallis, Oregon, United States

## Abstract

Daily interpersonal stressors, such as arguments with spouses, are predictors for increased negative affect within the literature (Witzel & Stawski, 2021). Emotion work, or activities done to promote another’s positive emotional state, has been noted to have mixed effects on health and well-being (Umberson et al., 2020) and emotion work is more prevalent in times of increased stress (Rao, 2017). Moreover, with old age, individuals experience fewer daily interpersonal stressors (Witzel & Stawski, 2021). Using the 2015 wave of the Health and Relationships Study (Ncouples=756, Mage=48.22, Rangeage=35-65, Mmaritalduration=8.52; Umberson, 2015), we explored 1.) how daily arguments with spouses within diverse marriages were related to negative affect across 10 days and 2.) whether age and emotion work moderate these associations. HARP is a daily diary study exploring marriage and health in same-sex and different-sex marriages. Preliminary results using multilevel models suggest that individuals’ negative affect significantly increased on days they reported spousal arguments. The interaction between spousal arguments and emotion work was significant (p<.05); on days when individuals reported more emotion work, negative affective reactivity associated with spousal arguments was higher, compared to days when less emotion work was reported. No significant age differences were found. Discussion will focus on the import of spousal arguments and emotion work for shaping daily well-being, and their relevance throughout adulthood, midlife, and older age. Future work should explore how being the recipient of emotion work may buffer or exacerbate the associations between daily arguments and negative affect and potential gendered patterning therein.

